# No direct by maternal effects interaction detected for pre-weaning growth in Romane sheep using a reaction norm model

**DOI:** 10.1186/1297-9686-45-37

**Published:** 2013-09-30

**Authors:** Ingrid David, Frédéric Bouvier, Edmond Ricard, Julien Ruesche, Jean-Louis Weisbecker

**Affiliations:** 1 INRA UR631 SAGA, Castanet-Tolosan F-31326, France; 2 INRA UE0332 Domaine de la Sapinière, Osmoy F-18390, France; 3 INRA UE0065 Domaine de Langlade, Montgiscard F-31450, France

## Abstract

**Background:**

The pre-weaning growth of lambs, an important component of meat production, depends on maternal and direct effects. These effects cannot be observed directly and models used to study pre-weaning growth assume that they are additive. However, it is reasonable to suggest that the influence of direct effects on growth may differ depending on the value of maternal effects i.e. an interaction may exist between the two components.

**Methods:**

To test this hypothesis, an experiment was carried out in Romane sheep in order to obtain observations of maternal phenotypic effects (milk yield and milk quality) and pre-weaning growth of the lambs. The experiment consisted of mating ewes that had markedly different maternal genetic effects with rams that contributed very different genetic effects in four replicates of a 3 × 2 factorial plan. Milk yield was measured using the lamb suckling weight differential technique and milk composition (fat and protein contents) was determined by infrared spectroscopy at 15, 21 and 35 days after lambing. Lambs were weighed at birth and then at 15, 21 and 35 days. An interaction between genotype (of the lamb) and environment (milk yield and quality) for average daily gain was tested using a restricted likelihood ratio test, comparing a linear reaction norm model (interaction model) to a classical additive model (no interaction model).

**Results:**

A total of 1284 weights of 442 lambs born from 166 different ewes were analysed. On average, the ewes produced 2.3 ± 0.8 L milk per day. The average protein and fat contents were 50 ± 4 g/L and 60 ± 18 g/L, respectively. The mean 0–35 day average daily gain was 207 ± 46 g/d. Results of the restricted likelihood ratio tests did not highlight any significant interactions between the genotype of the lambs and milk production of the ewe.

**Conclusions:**

Our results support the hypothesis of additivity of maternal and direct effects on growth that is currently applied in genetic evaluation models.

## Background

Pre-weaning growth is a complex trait for which phenotypic observations recorded on the lamb result from effects contributed by two individuals: by the lamb via direct effects and by the mother via maternal effects. The direct effects correspond to the suckling behaviour and growth ability of the young. Maternal effects depend on the mother’s ability to produce the milk required for growth and her maternal behaviour, and are strictly environmental from the perspective of the lamb [1]. Both direct and maternal effects are under genetic and environmental control. In the context of pre-weaning growth models, the standard assumption is that the observed phenotype is the sum of the environmental and genetic effects contributed by the ewe and its lamb [2]. In some cases, these models produce surprising results, e.g., strong negative estimates of the correlation between maternal and direct genetic effects [3,4]. Some authors have suggested that the hypothesis of additivity between direct and maternal effects may be too restrictive [5]. Experimental findings have demonstrated significant interactions between maternal and offspring genotypes in mice [6] and insects [7]. Wolf [8] gave a simple explanation of this in the case of mammals for growth performances: “…if offspring differ in how efficiently they process milk, and this difference has a genetic basis, then the contribution of the maternal character (performance on milk production) would not be additive”, or in other words, growth = milk production*feed conversion efficiency. This suggests the presence of an interaction between direct and maternal effects. Our aim was to test this hypothesis in Romane sheep in order to determine whether the assumption of additivity applied in current genetic evaluation models is appropriate or not. To achieve this, we developed an experimental design in which both growth and maternal phenotypes were recorded. Using a reaction norm model, we tested for the presence of an interaction between the direct genetic effect of the lamb and the maternal phenotypes.

## Methods

The experiment was carried out over a three-year period in order to obtain observations of maternal effects (milk quantity and quality) and growth records in Romane sheep. It consisted of mating ewes with markedly different maternal genetic effects (three groups: high, moderate and low maternal genetic effects) with rams that contributed very different direct genetic effects (two groups: high and low direct genetic effects) in four replicates of a 3 × 2 factorial plan. The measurements were performed on the lambs that resulted from these matings. Ewes and rams were selected from Romane sheep born at the INRA experimental farm La Sapinière (France) and which constitute the nucleus flock of the INRA401 composite sheep strain [9]. Estimated breeding values (EBV) based on average daily gain from 0 to 45 days (0-45 day ADG) obtained with an additive animal model (including direct and maternal effects) were used to classify the animals into different maternal and direct genetic effects groups. To obtain these EBV, data on lamb growth from 4260 multiparous Romane ewes (19 203 lambs, 294 sires) were analyzed. Random effects included in the model were correlated direct and maternal genetic effects and permanent environmental effects for the ewe and litter effects. Significant fixed environmental effects and one way interactions for age of the ewe, sex of the lamb, litter size at birth*litter size at weaning, and year*season were included also. Based on the resulting EBV, 240 multiparous ewes (2 to 3 years old) were selected: 80 with high maternal genetic effects, 80 with moderate maternal genetic effects and 80 with low maternal genetic effects. Twelve rams, six with low direct genetic effects and six with high direct genetic effects, were selected also. Animals were divided into two cohorts (named A and B), with equal numbers of animals from the direct and maternal genetic groups. During the experimental period, each ewe was mated twice (in April 2009 and April 2010 for animals in cohort A, in June 2010 and April 2011 for animals in cohort B). If the first mating of a ewe was performed with a male of the low direct genetic effects group, then the second mating was performed with a male of the high direct genetic effects group, and vice versa. The first mating of both cohorts was carried out at the La Sapinière experimental farm, after which the animals were moved to the INRA experimental farm Langlade for the second mating. Natural mating occurred after synchronization of the females by inserting a 20 mg FGA vaginal sponge (Chronogest CR, Intervet) for 14 days, followed by injection of 300 or 400 UI PMSG (Chronogest PMSG, Intervet) just after withdrawal of the sponge.

The lambs born from these matings were separated into two groups that were, respectively, artificially reared and maternally reared. In the maternally reared group, the lambs were twin-reared indoors with their mothers in small groups of six to nine ewes (eight groups per cohort and per year) in 14.5 m^2^ pens. From lambing to 28 days after lambing, ewes were fed with 900 g hay, 2700 g silage and 1100 g concentrate. From 28 to 42 days after lambing, ewes were fed with 1200 g hay, 3500 g silage and 650 g concentrate. To avoid stealing of milk, lambs were placed in a small pen when ewes were fed. Lambs did not have access to their mothers’ food until they were 35 days old. Lambs were weighed at birth, 15, 21, 35 and 60 days after lambing and at slaughter (between 90 and 120 days).

Milk yield was estimated using the lamb suckling weight differential technique [10] at around (depending on the date of lambing of the ewe) 15, 21 and 35 days after lambing, as follows: from 5:00 AM to 7:45 AM, lambs were separated from their mothers, then returned to the ewes and allowed to suckle and empty the udder. At 8:00 AM, they were once again separated from their mothers. At 10:30 AM, the ewes were moved to individual pens (1.45 m^2^) and the lambs were weighed prior to suckling the ewe for a short period lasting a maximum of 15 min (the lambs were removed from their mothers as soon as they finished suckling). The lambs were weighed again after suckling and lambs and ewes returned to separate pens. At 1:30 PM, the ewes were moved again to individual pens and the weigh-suckle-weigh procedure was carried out. After the second weighing, lambs and ewes were returned to the same pen. Milk composition was determined from samples of at least 15 mL collected by hand milking from both sides of the udder after a short lamb suckling period (2 minutes) in order to empty the cistern. The ewes and lambs were managed in the same way on the day of milk collection as on the day of milk yield evaluation. Samples were collected on days 15 and 21 at 10:30 AM and on day 35 after lambing at 10:30 AM and 1:30 PM and milk fat (MF) and protein (MP) contents were determined by infrared spectroscopy at the LIAL commercial laboratory. From day 35 postpartum, lambs were fed hay ad libitum and protein in the form of commercially-prepared lamb creep pellets.

Milk production records were used to define the maternal phenotypic effects of the ewes as a continuous variable. Two maternal effects were considered: milk yield per day (MY) and total milk solids (TMS), defined as TMS = (MF + MP)*MY for each of the three collection days (the mean MF and MP over the morning and afternoon were used to define milk composition on day 35). The ADG of maternally-reared lambs for the three periods (from 0 to 15, 15 to 21 and 21 to 35 days) were used as the dependent variable. In order to test whether there was an interaction between direct and maternal phenotypic effects (MY or TMS), the following recursive reaction norm model with known covariate [11,12] was used to analyse the data:

$$\begin{array}{l} \text{MILK}=\text{age}+\text{year}+\text{totsex}+\text{dam}\_\text{age}+v+\epsilon_{1}\\ \text{ADG}=\text{age}+\text{year}+\text{sex}+\text{dam}\_\text{age}+\text{WB}+\text{LW}\\ \;+\text{LSB}+\text{LW}*\text{age}+\beta \times \text{MILK}+u_{\text{int}}\\ \;+u_{\text{slope}}\times \text{MILK}+\epsilon_{2}, \end{array}$$

where *MILK* is *MY* or *TMS, ADG* is the ADG of the lambs for the three periods, *age*, *year*, *sex*, *WB*, *LW*, *LSB*, *totsex*, *dam_age* are the fixed class effects of age at weighing (15, 21 or 35 days), year (2009, 2010, 2011), sex of the lamb, weight of the lamb at birth (< 2.5 kg, > = 2.5 kg and < 3.5 kg, > = 3.5 kg and < 4.5 kg, > = 4.5 kg), lag in days between milk measurement and age at weighing ([-4,4]), litter size at birth ([2,5]), “sex” of the litter (1 = 2 females, 2 = 1 male and 1 female, 3 = 2 males), age of the dam (2, 3 or 4 years old); LW*age is the interaction between age at weighing and lag time between weighing and milk measurement, *β* is a regression coefficient , *v* is the additive genetic effect of the dam, *u*_*int*_ and *u*_*slope*_ are the additive genetic effects of the lamb on the intercept and slope of the linear reaction norms with variance covariance matrix $A⊗\left[\begin{array}{ccc} \sigma_{\mathrm{int}}^{2} & \sigma_{i‒s} & \sigma_{i‒v}\\ \sigma_{i‒s} & \sigma_{\mathrm{slope}}^{2} & \sigma_{s‒v}\\ \sigma_{i‒v} & \sigma_{s‒v} & \sigma_{v}^{2} \end{array}\right]$ , where *Α* is the additive relationship matrix, and *ϵ*_1_ and *ϵ*_2_ are residuals with variance covariance matrix $I⊗\left[\begin{array}{cc} \sigma_{\epsilon 1}^{2} & \sigma_{\epsilon 12}\\ \sigma_{\epsilon 12} & \sigma_{\epsilon 2}^{2} \end{array}\right].$ Fixed effects included in the models were selected in a step-by-step descending procedure, comparing the nested models with the likelihood ratio test (alpha risk was set at 5%). For this selection, single-trait models ignoring relationships were fitted using the mixed procedure of SAS version 8.1 [13].

The interaction between genotype of the lamb and milk production of the ewe was tested by comparing the previous recursive reaction norm model to a model that ignores the term *u*_*slope*_ x *MILK* (recursive intercept model), using the restricted likelihood ratio test (RLRT = -2logL_intercept_model_ + 2logL_reaction_norm_model_, where logL_x_ is the logarithm of the restricted maximum likelihood of model x),. These two models were fitted using the ASReml software [14].

In the recursive intercept model, heritability for MILK was estimated as $h_{\mathrm{MILK}}^{2}=\frac{\sigma_{v}^{2}}{\sigma_{v}^{2}+\sigma_{\epsilon 1}^{2}}$. The direct and maternal heritabilities for ADG were estimated as $h_{\mathrm{direct}}^{2}=\frac{\sigma_{\mathrm{int}}^{2}}{\sigma_{p}^{2}}$ and $h_{\mathrm{mat}}^{2}=\frac{\beta^{2}\sigma_{v}^{2}}{\sigma_{P}^{2}}$, with $\sigma_{P}^{2}\;=\;\sigma_{\mathrm{int}}^{2}+\sigma_{\epsilon 2}^{2}+\beta \left(2\sigma_{\epsilon 12}+\sigma_{i‒v}\right)+\beta^{2}\left(\sigma_{v}^{2}+\sigma_{\epsilon 1}^{2}\right).$

## Results

Heritabilities estimated with the additive model used to estimate EBV to select sires and dams for 0-45d ADG were 0.15 ± 0.02 and 0.07 ± 0.02 for direct and maternal genetic effects, respectively. The estimate of the genetic correlation between the two traits was 0.08 ± 0.14. The average maternal EBV for the low, medium and high maternal genetic effects group were -9.7 (standard deviation SD 4.4), 4.0 (SD 3.3) and 16.6 g/day (SD 3.5), respectively. The average direct EBV for the low and high direct genetic effects group were -14.1 (SD 8.5) and 29.1 g/day (SD 7.0), respectively.

The average fertility rate for the matings made was 79% and mean prolificacy was 2.4 lambs per ewe. There were 476 lambs in the maternally reared group (corresponding to 175 ewes, of which 71 naturally suckled their lambs during two successive years of the experiment). The average lamb mortality rate between days 0 and 60 was 2.5%. Thirty-four lambs were eliminated from the analysis because they were single suckling due to the death of its full-sib and were, therefore, not representative. Furthermore, 2% of the pre/post suckling weighing data were eliminated from the analysis because their lambs did not suckle for one of the time points. The final dataset contained 1284 records on 442 lambs born from 166 ewes.

The mean ADG of the lambs over the 0–35 day period was equal to 207 g/day (SD 46). As expected (Table 1), the mean ADG was significantly higher in the high than in the low direct genetic effects group (Δ = 12 ± 4 g/day) and in the high compared to the medium (Δ = 16 ± 5 g/day) and low (Δ = 28 ± 5 g/day) maternal genetic effects group.

**Table 1:** Mean (standard deviation) average daily gain from 0 to 35 days (g/day) for the direct and maternal genetic effects groups

		**Maternal genetic group**			
		**Low**	**Medium**	**High**	**Total**
Direct genetic group	Low	189 (35)	207 (49)	212 (38)	201 (42)
	High	198 (53)	209 (43)	229 (47)	213 (49)
	Total	193 (44)	205 (46)	221 (44)	207 (46)

Summary statistics on ADG and milk production by age are in Table 2. ADG was significantly higher in the 0–15 day period than in the 15–21 day and the 21–35 day periods (p < 0.0001). The average milk yield was 2.3 L/day (SD 0.8) and tended to decrease with age of the lambs over the 15–35 day period. There was a significant positive relationship between milk yield and the maternal genetic effects groups; the average milk yield was higher in the group with high maternal genetic effects (2.5 L/day) than in the groups with moderate (2.3 L/day) and low maternal genetic effects (2.2 L/day). The average MF was 60 g/L (SD 18) and the average MP was 50 g/L (SD 4). There was no clear difference in MF and MP between the maternal genetic effects group. The average TMS was 253 g/day (SD 87), significantly different depending on the maternal genetic effect group: means of 238 ± 5, 247 ± 5 and 272 ± 5 g/day in the low, medium and high maternal genetic group, respectively. Phenotypes for MY and TMS were, of course, highly correlated (0.90). The mean correlation between phenotypes for MY and ADG was 0.58 and remained quite stable with age of the lambs. The correlation between phenotypes for TMS and ADG was slightly lower (0.54).

**Table 2:** Average daily gain (ADG) of lambs and milk production (milk yield (MY) and total milk solids (TMS)) of the ewes on three days after lambing

**Day**	**ADG (g/day)**	**MY (mL)**	**TMS (g)**
15	223 (54)	2721 (734)	291 (83)
21	194 (52)	2358 (751)	253 (78)
35	194 (49)	1905 (752)	217 (84)

Standard deviations are in brackets.

The recursive reaction norm model with TMS as a covariate did not converge. Thus, results of models with TMS are not presented. Variance components estimated with the recursive intercept and reaction norm models with MY as covariate are in Table 3. Results of the restricted likelihood ratio test, comparing the recursive intercept and reaction norm models showed no significant interaction (RLRT_MY_ = 1.58) between direct genetic and maternal phenotypic effects. The residual variance was similar for both models (≈ 820) and much lower than the phenotypic variance (2887). Heritabilities estimated for the direct and maternal effects for ADG in the intercept model were higher than estimates from the additive model that was used to select parents of the offspring: 0.34 ± 0.04 and 0.23 ± 0.03, respectively. The heritability of MY was high 0.66 ± 0.03. The estimated genetic correlation between direct and maternal effects was high and positive at 0.47 ± 0.26. The regression coefficient linking ADG to MY was similar for both models (3.9), indicating that a 0.1 L/day increase in MY by the mother induced a 3.9 g/day increase in ADG for each of her lambs.

**Table 3:** Estimates of parameters obtained with the recursive intercept and linear reaction norm models for average daily gain of lambs with milk yield (dL) as covariate

	**Intercept model**	**Reaction norm model**
Δ(-2logL)	1.58	
$\sigma_{\epsilon 1}^{2}$	18	18
$\sigma_{v}^{2}$	35	35
$\sigma_{\epsilon 2}^{2}$	824	818
$\sigma_{\mathrm{int}}^{2}$	789	768
$\sigma_{\mathrm{slope}}^{2}$		126
*ρ*_*i* ‒ *s*_		-0.23 ± 0.30
*ρ*_*i* ‒ *v*_	0.47 ± 0.26	0.46 ± 0.27
*ρ*_*s* ‒ *v*_		-0.59 ± 0.94
*β*	3.9	3.9

Δ(-2logL) : restricted likelihood ratio test = -2logL_intercept_model_ + 2logL_reaction_norm_model_. $\sigma_{\epsilon 1}^{2},\sigma_{\epsilon 2}^{2},\sigma_{v}^{2},\sigma_{\mathrm{int}}^{2},\sigma_{\mathrm{slope}}^{2}$ are the variances of the residuals for MY and ADG, of the additive genetic effect of the dam on MY, of the additive genetic effects of the lamb on the intercept and slope of the linear reaction norms, respectively. *ρ*_*i* ‒ *s*_, *ρ*_*i* ‒ *v*_, *ρ*_*s* ‒ *v*_ are the correlations between intercept and slope, intercept and maternal effects, slope and maternal effects. *β* is the regression coefficient.

## Discussion

The mean prolificacy rate observed during our experiment was higher than that reported in Romane sheep by the French national recording scheme in 2010 (1279 ewes with a mean prolificacy rate after female synchronization equal to 2.13), in part because national records include younger females. Lamb mortality in the naturally-reared group (2.5%) was lower than that reported for the Romane breed in France (6%) but within the same range as that reported by another study in Scottish Blackface sheep [15]. Average growth rate was in line with that reported for the breed (French national recording scheme).

The objective of this study was to test whether an interaction exists between direct genetic and maternal phenotypic effects affecting ADG, given that these two components cannot be observed in practice. Environmental sensitivity to unobservable environmental factors can be analysed using a reaction norm model with an unknown covariate (RNUC). Different approximations [16] or ad-hoc procedures [17] have been reported to account for unknown covariates in reaction norm models. In RNUC, estimation of covariance functions is very sensitive to the data structure (number of animals per environment and connectedness between environments) [18]. Furthermore, Shariati et al. [19] demonstrated that the variance of the environmental effects is not identifiable if there is not at least one pair of unrelated animals in the same environment, which does not occur for pre-weaning growth, except with cross-fostering. For these reasons, we did not use RNUC to test for an interaction of direct with maternal effects but designed an experiment to obtain observations of the environmental effects contributed by the dam.

The experimental approach used is valid only if we are confident in the measurements of maternal effects obtained, i.e. that they are not biased. We evaluated milk yield using the weigh-suckle-weigh method. Since it had been shown that there is a positive correlation (0.86) between milk yield estimates obtained over a full 24 h period and those derived from a 12 h period [20], we extrapolated the daily yield using a 6 h test period for experimental convenience, similar to what has been done by other research groups [21]. It is acknowledged that the weigh-suckle-weigh method tends to underestimate milk yield, for several reasons: a lamb may not be able to consume all the milk available during a short period of suckling [10], a disturbance of normal suckling behaviour on the day of the test may induce a reduction in milk intake by the lamb [22] or inhibit milk release by the ewe [23], and the weight increment may be underestimated due to the voiding of faeces and urine [22]. In order to limit such underestimation, milk yield was only estimated in ewes rearing two lambs to ensure that all available milk was consumed, and the ewes and lambs were trained to be separated and grouped several times during the day. The excretion of faeces or urine between two successive weighings was recorded during a test of the weighing method and the proportion of such events was found to be small (less than 8% of the lambs). Also, underestimation of milk yield is not a problem in our case since it is a non-differential bias that does not affect the ranking of the ewes for milk yield. To obtain a more accurate measurement of milk production, one solution might be to make a third mating and to collect milk from the ewe by a milking machine throughout lactation (assuming that lactation remains stable with age). Milk production adjusted for the number of lambs born then corresponds to a maternal effect if there is no difference between ewes regarding how they accept the constraint of machine milking. However, milking is a difficult task in meat sheep.

To test the presence of an interaction between direct and maternal effects, we used a linear reaction norm model. Higher-order polynomials were tested but the models failed to converge. In the literature, reaction norm models with significant higher order terms are exceptions [24,25]. To take the relationship between ADG and milk yield into account, we used a recursive model, which assumed that milk yield of the ewe affected ADG of the lamb but that ADG had no effect on milk yield. Some authors have considered a simultaneous relationship between ADG and average daily feed intake [26]. This positive feedback of ADG on milk yield may not occur in our case because, as explained previously, twin rearing should insure that the maximal milk production potential of the ewe is reached regardless of the ADG of the lambs. The estimate of heritability for milk yield was higher than has been reported in the literature for dairy sheep [27]. Estimates of heritabilities for direct and maternal effects for ADG from the reaction norm model were also higher than reported in the literature [28] and than estimated with the additive linear model that was used to select parents of the offspring. Higher heritabilities can be explained by the selection of parents and the more controlled environment in which the study took place in comparison with field data which is confirmed by the lower ratio of the residual variance to the phenotypic variance observed on our experimental data in comparison with field data (0.28 versus 0.46). Furthermore, our reaction norm model did not include a permanent environmental effect of the lamb (the model did not converge for that case) to take repeated measurements into account, which could also have contributed to overestimation of the heritability. For MY, the permanent environment of the ewe was not included in the model because its variance was not significantly different from 0. Our estimate of the genetic correlation between direct and maternal effects was in the range of those obtained in previous studies, which varied from -0.52 [29] to +0.52 [30].

**Figure 1:**
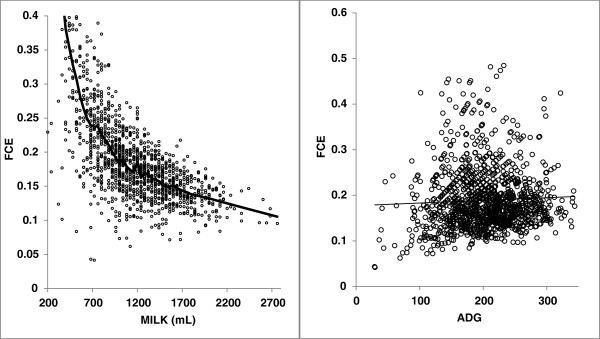
Change in feed conversion efficiency (FCE = milk consumed per day/average daily gain) with milk yield (MY) and average daily gain (ADG).

Contrary to what was expected, we were not able to identify a significant interaction between direct effects and milk production using the reaction norm model. A first explanation for the discrepancy between our findings and our interpretation of the physiological equation (growth = feed conversion efficiency*milk consumption*milk quality (physiology) ⇔ growth = direct effects* milk consumption*milk quality (interpretation)) could be that our test had insufficient power because, although parents were chosen based on extreme direct or maternal genetic effects, the genetic difference between direct effects for lambs or between milk productions of the ewes was not sufficiently large. A second explanation is that our interpretation of the physiological equation is correct but the feed conversion efficiency (FCE) varied with the milk consumed differently depending on the genotype of the lamb in such a way that it eliminated an interaction between direct effects of the lamb and milk production. The corresponding model for ADG of animal *i* in (milk) environment *j* could be the following (ignoring other environmental effects for the sake of simplicity): *ADG*_*ij*_ = (*μ*_*FCE*_ + *u*_*ij*_)*MY*_*j*_ + *ϵ*_*ij*_ (model 1) where *u*_*ij*_ is the direct genetic effect of animal *i* in environment *j* and *μ*_*FCE*_ is the mean feed conversion efficiency. If $u_{\mathrm{ij}}=\frac{u_{i}}{MY_{j}}$ then model 1 becomes *ADG*_*ij*_ = *μ*_*FCE*_*MY*_*j*_ + *u*_*i*_ + *ϵ*_*ij*_ , which is a model with no interaction between direct and maternal effects. Change in feed conversion efficiency with change in feed intake has been reported in the literature [31-34]. In our case, we observed that the FCE decreased with the quantity of milk consumed and was not correlated with ADG (Figure 1). Nonetheless, we found no strong evidence in the literature indicating that the effect of feed intake on FCE differs depending on the genotype of the animals considered. To confirm this hypothesis, it would be interesting to evaluate the FCE of lambs with different genotypes when they are artificially reared with different controlled quantities of milk available each day. We plan to conduct this experiment in the near future on the experimental farm of Langlade (France).

## Conclusions

This experiment, conducted over a three-year period with a large number of animals, made it possible to obtain information on the milk production and consumption of Romane sheep which, to our knowledge, has not been reported in the literature. The aim of this study was to measure direct genetic and maternal phenotypic effects to test for the existence of an interaction between them. We were not able to highlight any interactions between the direct and maternal effects in this experiment, which supported the hypothesis of additivity used in genetic evaluation models.

## Competing interests

The authors declare that they have no competing interests.

## Authors’ contributions

ID conceived and designed the experiments, performed statistical analysis and wrote the manuscript. ER set up the tools for data capture. JR validated the data. FB, JLW were responsible for recording data. All authors read and approved the final manuscript.
